# Use of model systems to understand the etiology of fragile X-associated primary ovarian insufficiency (FXPOI)

**DOI:** 10.1186/1866-1955-6-26

**Published:** 2014-08-13

**Authors:** Stephanie L Sherman, Eliza C Curnow, Charles A Easley, Peng Jin, Renate K Hukema, Maria Isabel Tejada, Rob Willemsen, Karen Usdin

**Affiliations:** 1 Department of Human Genetics, Emory University, 615 Michael St, Emory University, Atlanta, GA 30322, USA; 2 Washington National Primate Center, University of Washington, Seattle, WA, USA; 3 Laboratory of Translational Cell Biology, Department of Cell Biology, Emory University, Atlanta, GA, USA; 4 Department of Clinical Genetics, Erasmus MC, Rotterdam, The Netherlands; 5 Molecular Genetics Laboratory, Genetics Service, BioCruces Health Research Institute, Hospital Universitario Cruces, Barakaldo, Biscay, Spain; 6 Laboratory of Molecular and Cellular Biology, NIDDK, National Institutes of Health, Bethesda, MD, USA

**Keywords:** Primary ovarian insufficiency, Premature ovarian failure, Fragile X syndrome, Fertility, Repeat expansion disorder, CGG repeat

## Abstract

Fragile X-associated primary ovarian insufficiency (FXPOI) is among the family of disorders caused by the expansion of a CGG repeat sequence in the 5' untranslated region of the X-linked gene *FMR1*. About 20% of women who carry the premutation allele (55 to 200 unmethylated CGG repeats) develop hypergonadotropic hypogonadism and cease menstruating before age 40. Some proportion of those who are still cycling show hormonal profiles indicative of ovarian dysfunction. FXPOI leads to subfertility and an increased risk of medical conditions associated with early estrogen deficiency. Little progress has been made in understanding the etiology of this clinically significant disorder. Understanding the molecular mechanisms of FXPOI requires a detailed knowledge of ovarian *FMR1* mRNA and FMRP’s function. In humans, non-invasive methods to discriminate the mechanisms of the premutation on ovarian function are not available, thus necessitating the development of model systems. Vertebrate (mouse and rat) and invertebrate (*Drosophila melanogaster*) animal studies for the *FMR1* premutation and ovarian function exist and have been instrumental in advancing our understanding of the disease phenotype. For example, rodent models have shown that FMRP is highly expressed in oocytes where it is important for folliculogenesis. The two premutation mouse models studied to date show evidence of ovarian dysfunction and, together, suggest that the long repeat in the transcript itself may have some pathological effect quite apart from any effect of the toxic protein. Further, ovarian morphology in young animals appears normal and the primordial follicle pool size does not differ from that of wild-type animals. However, there is a progressive premature decline in the levels of most follicle classes. Observations also include granulosa cell abnormalities and altered gene expression patterns. Further comparisons of these models are now needed to gain insight into the etiology of the ovarian dysfunction. Premutation model systems in non-human primates and those based on induced pluripotent stem cells show particular promise and will complement current models. Here, we review the characterization of the current models and describe the development and potential of the new models. Finally, we will discuss some of the molecular mechanisms that might be responsible for FXPOI.

## Review

Fragile X-associated primary ovarian insufficiency (FXPOI) is among the family of disorders caused by the expansion of a CGG repeat sequence located in the 5' untranslated region (UTR) of the X-linked gene *FMR1*. About 20% of women who carry an allele with 55 to 200 unmethylated CGG repeats, called the premutation (PM) allele, develop hypergonadotropic hypogonadism and cease menstruating before age 40, a condition also known as premature ovarian failure (POF). The 20% contrasts with a rate of about 1% of the general population (for reviews, see [1-3]).

The term primary ovarian insufficiency (POI) encompasses both POF and occult indicators of ovarian function such as decreased levels of the anti-Müllerian hormone and increased levels of the follicle-stimulating hormone. As this entire spectrum, including the altered hormone profile is observed among women with the PM [4-9], the term ‘FXPOI’ is well suited [10]. Importantly, the proportion of women with the PM who manifest occult hormone indicators or clinical signs of ovarian dysfunction is unknown, as is the extent to which such indicators predict infertility or POF. This is a clinically significant gap as women with FXPOI can still become pregnant and may have a child with fragile X syndrome [11].

FXPOI is clinically significant. The most immediate and significant consequence of diminished ovarian function is reduced fertility [12,13]. The state of early estrogen deficiency leads to additional clinical consequences such as an increased risk of low bone density, earlier onset osteoporosis and bone fractures [14], impaired endothelial function [15], earlier onset of coronary heart disease [16], and increased cardiovascular mortality and overall mortality (e.g., [17,18]). In addition, women who have an early menopause are reported to suffer from more anxiety, depression, somatization, sensitivity, hostility and psychological distress than women with normal ovarian function [19].

We know very little about the mechanisms leading to FXPOI. It is well established that full mutation carriers, or those with an allele of >200 methylated repeats that leads to silencing of *FMR1*, do not suffer from ovarian dysfunction. Thus, the significant reduction of the *FMR1* protein product, FMRP, does not appear to be the culprit. There are important molecular attributes of the PM: with increasing repeat length, there are increasing *FMR1* transcript levels and decreasing FMRP levels [20-24]. As discussed in more detail below, many researchers have postulated that *FMR1* mRNA gain-of-function toxicity may underlie FXPOI, as is the case for the other PM-associated disorder, fragile X-associated tremor/ataxia syndrome (FXTAS) [25].

Not all women with the PM suffer from POF or occult indicators of ovarian dysfunction. Four factors have been investigated to explain the incomplete penetrance of POF among PM carriers: repeat length, skewed X-chromosome inactivation (XCI), background genes and smoking. First, there is a strong non-linear association between the penetrance of POF and repeat number. Women with mid-range PM repeats (approximately 70 to 90 repeats) have the highest risk for POF. Carriers of both smaller and larger PM repeat lengths also have an increased risk of POF compared to the general population, but not to the same extent as mid-range repeat carriers [7,13,26-28]. Second, skewed XCI may play a role in modifying the risk or severity of FXPOI, as *FMR1* is located on the X chromosome. However, no study has found evidence for skewed XCI based on samples from fresh blood among PM carriers with FXPOI [5,7,28-31]. Assuming that XCI in blood can be used as a proxy for the correct target tissue, one possible explanation for this observation is that the toxic effect of the PM acts during a stage in development when both X chromosomes are active. Third, studies have shown that the risk of POF depends not only on the PM allele, but also on other background genes [27,32]. Finally, smoking, a known risk factor for reducing the age at menopause, has been shown to have the same effect on women with the PM as it does on non-carriers [13,27].

In short, little is known about the etiology of FXPOI and the cause of its reduced penetrance and variable expressivity. The development and use of model systems to uncover the associated mechanism has just begun. The overall goal of this review is to describe these model systems and the initial steps taken to elucidate the mechanisms underlying the association between the *FMR1* PM and ovarian function. We will begin with a description of the current rodent model systems, which are the most mature in terms of their characterization of the effect of the PM. We will then describe new models that have the potential to advance the field.

### Rodent model systems: recapitulation of FXPOI

Only recently have *FMR1* mutation murine models been used to study ovarian function (Table 1). Published results for two PM mouse models [33,34] and unpublished studies for another (RKH *et al.*, unpublished), indicate their value in studying the etiology for FXPOI. A full mutation mouse model further implicates *FMR1* as having an important role in folliculogenesis [35]. Finally, the characterization of expression patterns of *FMR1* in the ovary of the rat shows the potential of this model in understanding the toxic effect of the PM [36]. Here we will review these models with respect to their ovarian phenotype to underscore their importance in future research on FXPOI.

**Table 1:** **Comparison of *FMR1*-related ovarian phenotypes among rodent model systems**

	**CGGnih **[33]	**YAC-TG296 **[34]	**CGGdut **[40]	**Full mutation/knockout **[35]	**Rat **[36]
Genetic background	C57BL/6	FVB/N	C57BL/6 and FVB/n	FVB129P2	Sprague–Dawley
Premutation repeat size	Approximately 130 repeats (variable)	(CGG)_9_AGG(CGG)_9_AGG(CGG)_72_	>100 repeats (variable)	n/a	n/a
Expression of WT	FMRP expression:	FMRP expression:	FMRP expression:	n/a	FMRP expression:
	• In GCs, LCs	• In GCs and oocytes at all follicle stages	• In GCs of growing follicles (20 weeks)		• In GCs, TCs, stroma of pre-antral follicles
	• In oocytes of all ages (high in primordial, primary and early pre-antral follicles)				
		• Not in interstitial cells			
					• LCs in older stages
			• In oocytes		
	• Not in interstitial cells	*FMR1* mRNA expression:			• In oocyte cytoplasm in primordial follicles (only observed in nucleus in a small fraction of cells)
		• In GCs and oocytes at all follicle stages			
	• Higher levels in ovaries compared with brain				
					• Not in stromal cells
		• Not in interstitial cells			• Weaker in atretic cells
					• Levels increased with increasing stage of follicular development
					*FMR1* mRNA expression:
					• Pre-antral and early antral follicles had higher expression than pre-ovulatory follicles
FMRP levels in model	• Reduced FMRP in GCs and LCs	No altered expression measured at 6 to 8 weeks	43% reduced in ovaries at 40 weeks		n/a
	• At 7 months, no altered levels in any ovarian cell				
	• At 7 months, abnormal distribution, higher in nucleus than cytoplasm				
*FMR1* mRNA levels in model	• Increased at all ages in ovary	Increased levels in ovaries	Increased in ovaries by 4.8-fold at 40 weeks		
	• Increased in GCs and oocytes (7 months)				
Nuclear inclusions	• One seen in 1 oocyte of 1/45 PM mice	Not done	None		
	• None observed in other ovarian cells				
Ubiquitination	• Higher levels in oocytes	Not done			
	• Animals with > FMRP nuclear distribution had highest number of oocytes with high concentration of ubiquitination				
Histopathology	• Grossly normal at 4 months, but 15% smaller	• Reduced uterine weight		• Increased number of follicles by 3 weeks	
	• Ovarian volume did not change from 4 to 12 months; no correlation with total number of oocytes and corpus lutea (in WT, declined linearly and strong correlation)	• At PD8 and PD25, ovary size did not differ			
		• Smaller size at 9 and 16 weeks		• Ovaries larger by 3 weeks (by 12 and 18 weeks 22% and 72% larger by mass than controls)	
	• Interstitial hypertrophy and tubulostromal hypertrophy (7 to 12 months)			• Prominent cysts consistent with corpus lutea development at 18 weeks	
	• Increase in ovarian cysts (incidence, number and size)				
Pattern of follicle counts compared with WT	• Same at 4 months	• At PD25, same number of primordial follicles			
	• >4 months, fewer follicles	• Number of mature follicles reduced at PD25 and 9 weeks			
	• All subclasses of follicles were reduced				
	• Size of primordial pool correlated with number of advancing subclasses	• Reduction of less mature follicle stages but not significant			
Corpus luteum characteristics	• Reduced	• Reduced at 9 weeks			
	• Decline associated with number of advanced follicles				
Granulosa cell characteristics	• Antral follicles had 15% fewer GCs	• Appeared with detached GC layer			
	• Corona of mature follicles was partial or missing leading to premature meiotic progression				
Atresia characteristics	• High ratio of atretic follicles to advancing follicles irrespective of estrus cycle	• Strong positive TUNEL in follicles at PD35 to 22 weeks showing an increased number of atretic cells			
	• Association of atresia rate and repeat number				
Fertility characteristic	• Not published, but unpublished work from same group supports a lower fecundity.	• Increased sterility			
		• Delayed time to first pregnancy			
		• Reduced number of pups per litter			
Hormone profile	Not done	• At 10 to 12 weeks, higher levels of 17β-E2, but similar levels at 16 and 22 weeks			
		• From 9 to 22 weeks, follicle-stimulating hormone higher and LH lower			
Expression of other genes	Not done	• At PD25, 8 and 14 weeks, LH receptor downregulated		• Increased protein levels of Tsc2, Sash1 and mTOR	
		• Large number of LH-induced ovulation-related genes downregulated at proestrus stage in adults			
		• Reduced phosphorylated Akt and phosphorylated mTOR, but total levels of Akt and mTOR were not altered			
		• No alterations in other major known regulators of folliculogenesis			

GC, granulosa cell; LC, luteal cell; LH, luteinizing hormone; n/a, not applicable; PD, postnatal days; PM, premutation; WT, wild type; TC, theca cells; TUNEL, terminal deoxynucleotidyl transferase dUTP nick end labeling; Akt, also known as Protein Kinase B (PKB), is a serine/threonine-specific protein kinase; mTOR, mammalian target of rapamycin.

#### ***Model construction***

The construction of each model has been reported in detail previously. Dr Usdin’s team originally constructed a knock-in (KI) murine model to study instability of the repeat sequence [37]. The approximately 130-repeat tract in the PM model was generated by serial ligation of short, stable CGG · CCG-repeat tracts, which were then used to replace the endogenous shorter murine repeat tract by homologous recombination. The KI allele had only minimal differences from the wild-type (WT) murine *Fmr1* gene in the region flanking the repeat. Therefore, females have a normal mouse *Fmr1* allele, and an *Fmr1* allele that is almost exactly the same as the endogenous mouse allele except for the length of the repeat tract. These mice are in a C57BL/6 background. This model will be referred to as CGGnih.

Lu *et al*. used a transgenic model that carries a YAC with the human PM allele that includes 90 repeats [34]. The line used (YAC-TG296) includes one copy of the YAC and about 5 kb of flanking sequence and was bred to WT FVB mice for five generations [38]. These mice are homozygous for the WT *Fmr1* allele. This line and several others have been used to study repeat instability [38] and the overexpression of FMRP [39].

Dr Willemsen’s team characterized their previously constructed KI mouse with an expanded CGG repeat in the PM range (CGGdut). This KI mouse model was developed by substituting the endogenous mouse 5' UTR containing the CGG repeat with the corresponding region from a human allele carrying 98 CGG repeats [40]. These mice are homozygous for the KI allele and have no WT *Fmr1* allele. They are on a mixed C57BL/6 and FVB/n genetic background. This model shows instability upon transmission [41] and the biochemical, phenotypic and neuropathological characteristics of FXTAS [42]. At this time, this model provides information about *FMR1* expression in ovarian tissues. Further work on the ovarian phenotype is currently being conducted.

The role of FMRP in ovarian function has also been examined in two other rodent models. Ovarian function in a mouse model for the fragile X full mutation containing a targeted disruption of the *Fmr1* gene [43] has recently been described [34]. The expression of FMRP and *Fmr1* during folliculogenesis has recently been evaluated in Sprague-Dawley rats [36].

#### ***Premutation leads to altered FMR1 expression levels***

In all WT animals, FMRP has been identified in granulosa cells (GCs), luteal cells and most prominently in oocytes. In oocytes, expression was seen at all stages of folliculogenesis and primarily in the cytoplasm [33,36]. Expression was not observed in the interstitial cells. For the rat model, Ferder *et al*. [36] found that there were changes in *Fmr1* expression during follicle maturation, both at the protein and mRNA levels. FMRP levels increased with increasing follicle development. *Fmr1* transcript levels were similar in pre-antral and early antral follicles, but decreased in pre-ovulatory follicles. The authors suggested that *Fmr1* expression in the ovary may be regulated at different levels and these may be independently controlled. In addition, they found expression of at least four different isoforms of FMRP during all stages of follicular growth. These expression patterns differ from those observed in the brain and testis.

Increased expression of *Fmr1* mRNA in the ovary has been seen in all PM mouse models. Interestingly, in the CGGnih and WT littermates, there was a non-linear age effect, where total ovary mRNA levels were higher at 12 months compared with 6 and 18 months of age. At 7 months, *in situ* hybridization studies of the CGGnih model showed mRNA levels to be increased in oocytes and GCs.

The expectation for relative FMRP levels differs among the PM mouse models due to their construction. No differences in FMRP levels between YAC-TG296 mice and their WT littermates were found, when measured at 6 to 8 weeks. At 1 to 2 months, the CGGnih mice showed relatively reduced levels of FMRP in GCs and luteal cells. In the CGGdut PM model, a reduction in FMRP expression was noted at 2 months. This observed decrease is similar to that found in the brain of these PM models [37,41]. Again, the relative *Fmr1* PM levels seemed more pronounced in ovaries than in the brain for both the CGGnih and the CGGdut models.

Interestingly, an abnormal distribution of FMRP when measured at 7 months has been observed in the CGGnih model: FMRP was more highly expressed in the nucleus of oocytes than in the cytoplasm. There were eight times as many oocytes with higher nuclear expression in the PM model compared with the WT.

Two phenomena considered to be a consequence of altered *FMR1* expression in the brain were measured for the CGGnih model: the presence of inclusion bodies and ubiquitination. Essentially no inclusions were noted in the ovarian cells of the PM or WT mice. With respect to ubiquitination, ubiquitin in WT mice was distributed throughout the cytoplasm and nucleoplasm. The CGGnih mice showed higher levels of ubiquitin in oocytes, more oocytes with elevated ubiquitin and a pronounced nuclear/perinuclear concentration than WT mice. Also, those with the highest number of oocytes with nuclear FMRP had the highest number of oocytes with high concentrations of ubiquitin.

#### ***Morphology of the premutation ovary***

Both the CGGnih and YAC-TG296 models had smaller ovaries by 4 months of age compared with WT mice, but they were grossly normal. However, in the CGGnih model from 4 to 12 months, there was no decrease in size, as would be expected with the normal decrease in the number of oocytes and corpus lutea. This might be explained by the noted interstitial hypertrophy and tubulostromal hypertrophy at 7 to 12 months. The CGGnih mouse ovaries also had more and larger non-functional ovarian cysts.

#### ***Premutation leads to a depletion of follicles in later stages of maturation***

Examination of the pattern of follicle counts at all stages provides insight into the effect of the PM. The total number of primordial follicles was comparable to WT measured at PD25 (YAC-TG296) and at 4 months (CGGnih). This suggests that the establishment of the primordial pool is not affected in PM mice. At PD25 and 9 weeks in the YAC-TG296 model, the number of later subclasses of follicles was reduced, and significantly so for mature follicles, compared with WT mice. At ages over 4 months, the CGGnih mice had a significant reduction of all subclasses of follicles, with the size of the primordial pool being correlated to the number of advancing subclasses. Also, the number of corpus lutea, the bodies that result from post-ovulatory follicles, was reduced in PM mice compared with WT mice. Together, these observations suggests that the PM does not affect the establishment of the primordial follicle pool, does not block a particular stage of follicle development and does not lead to increased follicular recruitment. The fact that both follicles that depend on ovarian intrinsic factors and those that depend on the input of extrinsic factors are affected suggests that the problem may be intrinsic to the ovary.

#### ***Premutation leads to granulosa cell abnormalities***

GCs are key to the functioning of the follicle. The CGGnih mice had fewer GCs in the antral follicles than did the WT mice. Furthermore, there were significantly more antral follicles in which the GC layer was detached and the corona was partial or missing in both the CGGnih and YAC-TG296 models. Signs of atresia were also increased. In the CGGnih mice, there was a high ratio of atretic follicles to advancing follicles, irrespective of estrus cycle stage. Using terminal deoxynucleotidyl transferase dUTP nick end labeling (TUNEL) to analyze ovarian sections at PD35, 16 weeks and 22 weeks, the YAC-TG296 mice were seen to have increased numbers of antral follicles that appeared atretic compared with the WT mice. Thus the PM could lead to increased apoptosis in the ovaries.

#### ***Premutation leads to subfertility***

Features of fertility were investigated for the YAC-TG296 mice. These mice had an increased frequency of sterility, and, among those that were fertile, reduced litter sizes and were older in having their first litter. At 9 to 22 weeks, these mice had higher follicle stimulating hormone and lower luteinizing hormone levels compared with WT mice. They also had higher levels of 17β-E_2_ at 10 to 12 weeks, although these levels normalized to those of WT mice at 16 to 22 weeks.

#### ***Premutation leads to altered gene expression***

Expression of genes known to be involved in ovarian function was investigated for YAC-TG296 mouse ovaries at two stages: PD25 and proestrus-stage adults (8 to 14 weeks). The LH receptor (Lhr) was significantly downregulated at both stages. However, no differences in mRNA levels were found between PM and WT mice, among other major known regulators and markers of folliculogenesis. LH-induced ovulation-related genes were further investigated and found to be downregulated, specifically at the proestrus stage in adults. These findings suggest that the LH-mediated pathway could be affected in PM ovaries. The PI3K-Akt pathway, a pathway known to play a critical role in gonadotropin-mediated GC differentiation, cumulus expansion and oocyte maturation, was also investigated. YAC-TG296 mouse ovaries had a significant reduction in the levels of phosphorylated Akt, but not total Akt. Given the interaction between the Akt and mTOR pathways, the status of mTOR was also investigated. Again, there was a reduction in the levels of the phosphorylated mTOR protein but not total mTOR. Thus, the Akt-mTOR-mediated signaling cascade may be altered in PM ovaries. A role for reduced mTOR phosphorylation in FXTAS is suggested by the observation that activating mTOR ameliorates neurodegeneration in a fly model of FXTAS [44]. It will be of interest to see whether this activation improves ovarian function in flies and mouse models.

#### ***Ovarian phenotype altered in knockout model***

An interesting ovarian phenotype has been observed in the full mutation knockout (KO) mouse model [35]. By 3 weeks, homozygous KO mice had an increased number of follicles compared with WT mice. By 18 weeks, the size of the ovaries in the KO mice was larger than those in the WT mice and showed prominent cysts, consistent with corpus lutea development. Lysates from 9-to-18-week-old ovaries showed increased protein levels of Tsc2, Sash1 and mTOR. The authors suggested that increased levels of these proteins seen in the absence of *FMRP*, may lead to precocious follicular development. Thus, this KO model may have the potential to model ovarian insufficiency; however whether the related mechanism is associated with FXPOI is an open question. Women who carry the full mutation do not show signs of POI. Whether this is due to the fact that they are heterozygous for the loss of FMRP is unknown.

### Fly model: effect of modifying genes and more

In *Drosophila* ovaries, a small population of germ-line stem cells (GSCs) is maintained in a well-defined microenvironment. This provides an attractive system for investigating the regulatory mechanisms that determine the fate of stem cells [45,46]. A typical *Drosophila* ovary is composed of 16 to 20 ovarioles. Each ovariole consists of an anterior functional unit called a germarium that houses GSCs and somatic lineages, and a linear string of differentiated egg chambers posterior to the germarium. The tip of the germarium consists of specialized cells that maintain the microenvironments called niches and these are essential for GSC proliferation and maintenance. At this tip, GSCs normally divide asymmetrically to ensure that one daughter cell remains attached to the niche cells for self-renewal, while the other is displaced from the niche, becoming a cystoblast, which initiates differentiation and sustains oogenesis [47]. Studies from multiple laboratories have identified the genes that are essential for GSC fate determination [48,49].

*Drosophila* GSCs have been used as a model to show that FMRP can modulate the fate of stem cells: Yang *et al*. [50] found that *dFmr1* is required for both maintaining GSCs and repressing differentiation. Very recently, transgenic lines have been developed that drive the expression of the PM rCGG repeat in fly ovaries and these rCGGs were found to be toxic in the germ line as well (PJ, unpublished data). These results suggest that both the reduction of FMRP and expression of PM rCGG repeats could have detrimental effects on fly ovary and stem cell maintenance.

Because of the relative ease of model construction compared with other model systems, two other important questions can be addressed at relatively low cost. First, the fly model can be used to test the effect of genetic modifiers on the ovarian phenotype. This could be valuable not only for our understanding of the pathogenic mechanism, but may also shed light on genes whose human homologs may contribute to the variable penetrance of FXPOI. Second, the *Drosophila* model has significant potential for increasing our understanding of the non-linear effect of repeat number by creating constructs that vary only by repeat number.

### Non-human primate model: bridging the translational gap

Many genetic, cellular and physiological differences exist between the current model systems used for studying FXPOI and human females. Non-human primates (NHPs) offer a clinically relevant model system in which to explore the molecular mechanisms of the PM on ovarian function. One of the limitations in modeling FXPOI is that there are no known naturally occurring animal models with *FMR1* repeat mutations, including NHPs [51,52]. Of the species tested thus far, only NHPs have CGG repeat numbers comparable to those in humans [53]. The repeat sequence found in the great apes (Hominidae) shows striking similarity to that in humans, with CGG repeat lengths ranging from 20 to 39 interrupted by 1 to 6 AGG interruptions and the longest and most variable CGG lengths at the 3' end of the repeat [53,54].

While it is possible that spontaneous CGG repeat expansion to the pre- and full mutation range does occur in NHP populations, screening would be expensive and unlikely to yield sufficient animal numbers for meaningful studies. Instead efforts are currently underway at the Washington National Primate Research Center to generate a NHP transgenic model of FXPOI. Using technologies based on embryonic stem cells, Dr Curnow’s team aims to introduce the human PM sequence into the macaque endogenous *FMR1* gene and generate NHP females with germ-line expression of the PM. While embryonic stem cells from species other than the mouse have been historically less amenable to gene-targeting strategies, recent work on the rat, human and marmoset has shown transgene efficiencies and stability equivalent to the mouse following the refinement of the culture conditions for embryonic stem cells and gene-targeting methods [55-62]. Full-scale development of a NHP model of FXPOI necessitates a long-term approach with the generation of a self-sufficient breeding colony of FXPOI-affected females in which reproductive function related specifically to FXPOI can be studied. These studies can be done in conjunction with other pertinent aspects of fragile X-associated disorders.

### Induced pluripotent stem cell model: examination of affected tissues

Findings from the PM mouse models noted above suggest that GC function is involved in the cellular cause of FXPOI, as is true for other forms of POI [63-65]. It is difficult to study GC function in women, as the procedure to obtain follicles with GCs is quite invasive and, thus, patient material is scarce. Patient-specific induced pluripotent stem cells (iPSCs) derived from adult somatic cells and which have differentiated into GC-like cells represent one novel possible option for generating an abundance of material for research purposes without any invasive procedures.

Work by Kang *et al*. has shown the ability of mouse iPSCs to differentiate into GC-like cells that express FSHR and secrete estradiol after co-culture with mouse GCs isolated from stimulated follicles [64]. However, this research has not been extended to human iPSCs. Adapting this protocol would enable researchers to investigate repeat length instability, cellular and signaling defects and cell viability in *in vitro* GCs derived from patient-specific human iPSCs. These types of *in vitro* studies could elucidate novel defects in somatic cells that support follicle survival and maturation that contribute to POI.

Recently, Hayashi *et al*. showed that functional oocytes could be derived from mouse iPSCs [66]. While this differentiation method relied on *in vivo* co-culture with normal mouse GCs transplanted under the ovarian bursa, the technique showed the feasibility of reconstituting a follicle and generating a functional oocyte from mouse iPSCs. If this system could be adapted to human female iPSCs using a wholly *in vitro* methodology in combination with a GC differentiation protocol, researchers could study how signaling defects in GCs contribute to oocyte death in POI. For treatment of infertility related to POI, patient-specific iPSCs could be differentiated into functional oocytes with GCs from non-POI patients to allow POI women to produce their own genetic offspring. Although these types of experiments and clinical implications are years away from being realized, human POI iPSC studies are a novel way for furthering understanding of FXPOI and its consequences.

### Possible mechanisms of FXPOI: what have we learned from model systems?

As emphasized above, we know little about the disease pathology underlying FXPOI. A number of lines of evidence suggest that the pathology is not related to an FMRP deficit. Firstly, FXPOI is seen in women who have repeat numbers that are not associated with an FMRP deficit, at least in peripheral blood. Secondly, women who carry the completely silenced full mutation, and thus do not express FMRP in, on average, half their cells, do not show symptoms of FXPOI. Thirdly, the YAC-TG296 mouse model is homozygous for the WT *Fmr1* allele, yet shows signs of ovarian dysfunction. Thus FXPOI, like FXTAS, is not likely to be the result of the loss of FMRP. However, how this relates to the observation that *Fmr1* KO mice also show ovarian dysfunction is unclear.

In terms of the molecular mechanism, there may be parallels to FXTAS. Work with tissue cultures has shown that expression of mRNA from the PM allele is deleterious to a wide variety of cell types; thus it is reasonable to think that FXTAS and FXPOI may share a common pathological basis. A variety of models have been proposed to explain the pathology of FXTAS and support for these comes from various model systems. The RNA gain-of-function model predicts that the long rCGG track sequesters specific CGG-binding proteins, resulting in the loss of normal cell function. Various proteins have been identified that directly bind to CGG-RNA and whose sequestration may affect cell viability, including: hnRNP A2/B1, a protein involved in pre-mRNA processing [67,68]; Pur α, a protein that has been implicated in transcription regulation and neuronal development [67]; and the miRNA-processing complex, DROSHA-DGCR8 [69]. These proteins in turn are able to recruit additional proteins like CUGBP1 in the case of hnRNP A2/B1 [70] and the RNA helicase, Rm62, in the case of Purα/DDX5 [71]. Overexpression of DROSHA [69], hnRNP A2/B1, CUGBP1 [72], Pur α [67] and Rm62 [71] rescue neurodegeneration in a fly model of FXTAS, but whether they rescue the mammalian phenotype remains to be seen.

Transcripts from the *FMR1* locus may be deleterious in other ways. For example, the rCGG forms stable secondary structures including hairpins [73,74], which are substrates for the human enzyme Dicer [74]. Dicer is responsible for generating small RNAs that can act via the RNA interference pathway to reduce post-transcriptionally the expression of genes containing similar repeat tracts. Expression of RNA with 80 CAG repeats, which also forms hairpins, generates Dicer-dependent small RNAs that are toxic to neuronal cells in culture [75]. It remains to be seen whether rCGGs could be toxic in similar ways.

Various antisense transcripts are also made from the human *FMR1* gene and these potentially contribute to disease pathology in different ways. While the expression of some of these transcripts is low in normal cells, in PM carriers some of these transcripts are present at levels comparable to that of the sense transcript [76]. Some double-stranded RNAs, including rCUG.rCAG, can activate the innate immune response in *Drosophila* in a Dicer-dependent manner [75]. Such double-stranded RNAs could be generated via the annealing of sense and antisense transcripts produced from the *FMR1* gene. The antisense rCGGs may also sequester proteins, as proposed for the sense transcript. Furthermore, since the repeat is located in a putative open reading frame on some of the antisense transcripts, it could produce a repeat-containing protein, in this case a polyproline-containing protein, which could contribute to disease pathology [76].

Another protein-based model arises from the observation that repeated sequences can increase the frequency at which translation initiates at non-ATG codons, a process known as repeat-associated non-ATG (RAN) translation [77]. In humans, and in mice and flies containing the human 5' UTR, there is evidence to suggest that such start sites are used to make polyglycine and/or polyalanine-containing proteins that are neurotoxic [78]. Such proteins can be detected in the brains of individuals with FXTAS.

The YAC-TG296 and the CGGdut mouse models do have the human *FMR1* 5' UTR upstream of the repeat and thus can make the polyglycine and polyalanine proteins. In contrast, the CGGnih mouse retains the murine 5' UTR and thus has a stop codon immediately upstream of the repeat. The fact that the CGGnih mouse does exhibit signs of ovarian dysfunction suggests that at least some pathology may arise independently of RAN translation. However, the relative contribution of RNA-based pathology and protein-based pathology to the overall phenotype remains to be determined.

Many diseases caused by expansion of different repeats are associated with the formation of intranuclear inclusions in patients, in cells in tissue culture as well as in the brains of mice and fly models. While intranuclear inclusions in the brain are a hallmark of FXTAS, very few inclusions are seen in the ovaries of PM mouse models and humans. More data are necessary to establish definitively whether inclusions are a feature of FXPOI. The few inclusions noted in stromal cells of grossly normal appearing ovaries from humans [79] may suggest an underlying toxic gain-of-function related to protein degradation. The absence of inclusions in follicles may be the result of the rapid loss of affected follicles, too fast for inclusions to be observed [79]. This would be similar to that seen in Purkinje cells in FXTAS [80]. However, it is unclear whether intranuclear inclusions are protective, pathogenic or neutral markers of disease pathology.

Lastly, more work needs to focus on altered gene expression in PM models. Data from the YAC-TG296 model shows reduced expression of phosphorylated Akt and mTOR, while the KO model showed elevated mTOR levels. mTOR dysregulation in these animals is of interest since both underexpression and overexpression of mTOR [81,82] can result in ovarian dysfunction. It has been shown that mTOR inhibition results in reduced GC proliferation [83], a significant phenotype in the PM mouse models. We speculate that a role for reduced mTOR phosphorylation in FXPOI could also account for the non-linear relation between CGG repeat number and the risk of FXPOI. As the repeat number increases, FMRP levels are predicted to decrease, because of the difficulties associated with the translation of large PM alleles. This decrease in FMRP could in turn lead to increased levels of mTOR phosphorylation, which could offset the loss of mTOR resulting from the consequences of expression of the PM rCGGs. However, whether mTOR dysregulation is the proximal cause of the ovarian dysfunction seen in FXPOI still remains to be determined and studies to address mTOR levels in human female PM carriers are sorely needed. Also, some phenotype differences observed between models may be related to the different background strains. Although this can complicate comparisons, it also points to the importance of modifying genes to explain the variable presentation of FXPOI.

## Conclusions

Clearly, the value of model systems in determining the underlying cause of FXPOI cannot be overstated. Each system has its advantages. The fly model will be valuable for determining the non-linear effect of increasing repeat number on the ovary in a cost-efficient way. It will also be the model of choice for identifying modifier genes using effective genetic screens. As always, caution must be taken in ascribing phenotype outcomes in the fly to mammals. In particular, the development and aging processes occurring within ovaries differ between the fly model and mammalian systems. Thus, disease progression and histological studies of FXPOI will need to be studied in mammalian models. Already rodent models have shown their potential and will continue to help in elucidating mechanisms and identifying potential treatments. Still, the genetic, cellular and physiological differences between current vertebrate model systems and humans suggest that additional model systems should be developed and tested in parallel to expedite translational research efforts. The translational bridge between current animal models and humans can best be met by NHP studies. Finally, patient-specific iPSCs derived from adult somatic cells and differentiated into GC-like cells represent a viable option for generating material needed for research without invasive procedures that minimizes the excessive use of animals. Thus, the combined use of model systems promises to elucidate the underlying mechanisms of FXPOI and the associated risk factors.

## Abbreviations

FXPOI: fragile X-associated primary ovarian insufficiency; FXTAS: fragile X-associated tremor/ataxia syndrome; GC: granulosa cell; GSC: germ-line stem cell; iPSC: induced pluripotent stem cell; kb: kilobase; KI: knock-in; KO: knockout; miRNA: microRNA; NHP: non-human primate; PM: premutation; POF: premature ovarian failure; POI: primary ovarian insufficiency; RAN: repeat-associated non-ATG; UTR: untranslated region; XCI: X-chromosome inactivation; WT: wild type; YAC: yeast artificial chromosome.

## Competing interests

The authors declare that they have no competing interests.

## Authors’ contributions

Each author contributed sections of the review that relate to their specific expertise in model systems and/or FXPOI. All were involved in the writing and editing of the final manuscript. All authors read and approved the final manuscript.
